# Magnetic Resonance Image under Variable Model Algorithm in Diagnosis of Patients with Spinal Metastatic Tumors

**DOI:** 10.1155/2021/1381274

**Published:** 2021-08-16

**Authors:** Hongliang Chen, Biao Xie, Xin Zhong, Xiang Ma

**Affiliations:** ^1^Department of Orthopaedics, Affiliated Hospital of Xuzhou Medical University, Xuzhou 221000, China; ^2^Department of Orthopaedics, The Third Affiliated Hospital of Kunming Medical University (Tumor Hospital of Yunnan Province), Kunming 650000, China; ^3^Department of Dermatology, Xuzhou Central Hospital, Xuzhou 221000, China

## Abstract

The aim of this study was to explore the adoption of the variable model algorithm in magnetic resonance imaging (MRI) image analysis and evaluate the effect of the algorithm-based MRI in the diagnosis of spinal metastatic tumor diseases. 100 patients with spinal metastatic tumors who were treated in hospital were recruited as the research objects. All patients were randomly divided into the experimental group (MRI image analysis based on variable model) and the control group (conventional MRI image diagnosis), and the MRI of the experimental group was segmented using the conventional algorithm with variable model and the improved algorithm with GVF force field. The accuracy index (Dice coefficient *D*) values were used to evaluate the vertebral segmentation effect of the improved variable model algorithm with the introduction of GVF force field, and the recognition rate, sensitivity, and specificity indexes were used to evaluate the effects of the two algorithms on the recognition of MRI image features of spinal metastatic tumors. The results showed that the mean *D* value of the variable model improvement algorithm for the segmentation of five vertebrae of spinal metastatic tumors was significantly improved relative to the conventional variable model algorithm, and the difference was statistically significant (*P* < 0.05). At the number of 80 iterations, the recognition rate, sensitivity, and specificity of MRI image segmentation of the traditional variable model algorithm processing group were 89.32%, 74.88%, and 86.27%, respectively, while the recognition rate, sensitivity, and specificity of MRI image segmentation of the variable model improvement algorithm processing group were 97.89%, 96.75%, and 96.45%, respectively. The results of the latter were significantly better than those of the former, and the differences were statistically significant (*P* < 0.05); and the comparison of MRI images showed that the variable model improvement algorithm was more rapid and accurate in identifying the focal sites of patients with spinal metastases. The accuracy of MRI images based on the variable model algorithm increased from 69.5% to 92%, and the difference was statistically significant (*P* < 0.05). In short, MRI image analysis based on the variable model algorithm had great adoption potential in the clinical diagnosis of spinal metastatic tumors and was worthy of clinical promotion.

## 1. Introduction

Spinal metastatic tumor is a common spinal disease at present. The clinical symptoms include vertebral bone destruction and collapse, pathological fractures, kyphosis, and severe spinal cord and nerve compression. Once the spinal nerve is damaged, its function will be difficult to recover [1, 2]. Therefore, the early accurate identification, diagnosis, and treatment of spinal metastatic tumors are of great significance to reduce deformities, prevent permanent nerve damage, and improve the quality of life of patients. At present, imaging-guided lesion biopsy is the gold standard for preoperative differential diagnosis of spinal metastatic lesions [3]. However, there are limitations in time, conditions, and so forth, and there are risks of damage to the spinal cord and nerves, as well as the possibility of false negatives, and it cannot be used in large-scale clinical adoptions. Therefore, the technical upgrade of early diagnosis of spinal metastatic tumors is a problem that needs to be solved urgently. The current diagnostic imaging methods for metastatic tumors of the spine include conventional radiographs, nuclear scans, CT, and MRI [4]. Among them, conventional radiographs can only detect bone deformation or compression fracture changes, which are not sensitive to metastatic tumors; the nuclear concentration phenomenon of bone lesions in nuclear scans makes the results lack specificity and be difficult to diagnose qualitatively [5]; CT has radiation and radiation artifacts in the nearby bone cortex [6], MRI has the advantages of no ionizing radiation damage, better tissue resolution than CT, and ability to be scanned in multiple directions in the transverse sagittal corridor. It is sensitive to metastatic changes in the spine and is gradually becoming the preferred method for the diagnosis of spinal metastases in clinical practice [7–9].

Currently, segmentation algorithms for medical images emerge endlessly, and the adoption of variable model algorithm in MRI image analysis has been mentioned in many reports. A variable model is a model that uses internal and external forces to push curves or surfaces in the image domain to move relative to one another within a certain range [10]. The internal force controls the model to remain smooth during the deformation process, while the external force pushes the model to move in the specified direction. Since the deformation model allows the integrated boundary factors to perform continuous operations on the image noise and boundary gaps on the continuum, the accuracy of the image edge area reaches the subpixel level, thereby improving the image quality [11, 12]. The variable model integrates information such as the location, size, and shape of the target area when processing medical images. The degree of fit between the medical image and the variable model is regulated by the principle of energy function minimization so as to complete the clear positioning of the location of the lesion in the image [13]. The current research showed that the variable model algorithm with active contour (Snake) as the key parameter presents a good segmentation effect in MRI image segmentation of brain tumors [14]. However, there is no report about the adoption of variable model algorithm in MRI image analysis of spinal metastatic tumors. Therefore, it was hoped to design an improved variable model algorithm for MRI image analysis of spinal metastatic tumors and evaluate the optimization effect of improved variable model algorithm in image segmentation by comparing it with traditional variable model algorithm in terms of image segmentation effect and focus positioning accuracy. Then, comprehensive evaluation of the degree of conformity of the improved variable model algorithm compared with the diagnosis results of traditional MRI image manual analysis was made through the comparative analysis of group diagnosis results and postoperative pathological results so as to judge the adoption value of the algorithm in the MRI imaging diagnosis of clinical spinal metastatic tumors.

## 2. Materials and Methods

### 2.1. Research Objects

In this study, 100 patients with spinal metastatic tumors admitted to hospital were selected as the research objects. Among them, 61 were males and 39 were females, ranging in age from 23 to 71 years, with an average age of 48.7 years. There were 35 cases in the cervical segment, 29 cases in the thoracolumbar segment, and 36 cases in the thoracic segment. The study had been approved by the ethics committee of the hospital, and the patients and their families included in the study had known the situation and had signed the informed consent. MRI scans were performed on all patients, and the patients were divided into experimental group (MRI image analysis diagnosis based on variable model algorithm) and control group (conventional MRI image analysis diagnosis) by random grouping. The number of cases in each group was 50.

Inclusion criteria were as follows: (i) patients were diagnosed with spinal metastatic tumors by postoperative pathological examinations; (ii) basic clinical data were complete; and (iii) preoperative MRI images were clear. Exclusion criteria were as follows: (i) those with incomplete clinical data and (ii) those who were not diagnosed with spinal metastatic tumors by postoperative pathological examinations.

### 2.2. MRI Scanning

MRI examinations were performed on all patients, and sagittal T1WI, T2WI, cross-sectional TIWI, and gradient recalled echo (GRE) serial sagittal scans were selected. MRI detection was performed via Anke ASM-015P MRI system and the surface coil of the neck and spine. The imaging included SE sequence conventional, coronal, sagittal, or axial T1 weighted (TR/500-TE/30 ms), T2 weighted (TR/1500-TE/90 ms), and GRF sequence (GR500/TE19/FA38) sagittal scan.

### 2.3. Construction of MRI Image Analysis Model for Spinal Metastatic Lesions Based on Variable Models

Using variable model algorithm to process MRI images is of great adoption value in clinical tumor diagnosis, which can greatly reduce the degree of dependence on the subjective judgment of physicians. The traditional variable model method defines a closed curve (closed surface in the case of 3D) in the image area. The initial envelope can move to the target contour under the action of internal and external forces. When it moves to the target contour, the envelope energy is the smallest. The internal force that pushes the envelope to move is determined by the smoothness and curvature of the envelope, and the external force is determined by the characteristics of the image target. The mathematical model expression is as follows:

(1) $$\begin{array}{c} E_{\text{snake}}=\int_{0}\frac{1}{2}\left\{\alpha |Y^{\prime }{\left(z\right)}^{|2}+\beta |Y^{\prime \prime }{\left(z\right)}^{{}^{|2}}+E_{O}\left(Y\left(z\right)\right)\text{dz}\right\}. \end{array}$$

In equation (1), *α* is the weight coefficient that controls the degree of smoothness of the dynamic envelope and *β* represents the weight coefficient that controls the degree of curvature of the dynamic envelope. The larger *α* is, the stronger the smoothness of the envelope is and the stronger the tensile capacity is. The larger *β* is, the stronger the bending resistance of the envelope is. If *β*=0, it means that the envelope can converge at the corner. As the energy potential function of the envelope, *E*_snake_ is composed of the sum of internal energy and external energy. For the classic grayscale image of MRI image (denoted as *N*(*a*, *b*)), when the target contour is at the edge of the step, the calculation equation of external energy is expressed as follows:

(2) $$\begin{array}{c} E_{O}^{1}\left(a,b\right)=-|\nabla {N\left(a,b\right)|}^{2}, \end{array}$$

(3) $$\begin{array}{c} E_{O}^{2}\left(a,b\right)=-|\nabla {\left(H_{m}\left(a,b\right)N\left(a,b\right)\right)|}^{2}. \end{array}$$

In equation (3), *H*_*m*_(*a*, *b*) represents the Gaussian function with two-dimensional standard deviation of *m*, and ∇ represents the gradient operator. When the image background is white and the image is black, the external energy is expressed as follows:

(4) $$\begin{array}{c} E_{O}^{3}\left(a,b\right)=N\left(a,b\right), \end{array}$$

(5) $$\begin{array}{c} E_{O}^{4}\left(a,b\right)=H_{m}\left(a,b\right){}^{\prime }N\left(a,b\right). \end{array}$$

According to equations (4) and (5), the larger *m* is, the more blurred the edge of the MRI image is. However, a relatively larger *m* should be selected to expand the range of external force in the MRI image processing for spinal metastatic tumors, based on which the variational method is used to minimize equation (1), and Euler's equation is obtained, which is expressed as

(6) $$\begin{array}{c} \alpha Y^{\prime \prime }\left(z\right)-\beta Y^{\prime \prime \prime \prime }\left(z\right)-\nabla E_{O}=0. \end{array}$$

The balanced form of the introduced force is expressed as follows:

(7) $$\begin{array}{c} F_{I}+F_{O}=0,\\ F_{I}=\alpha Y^{\prime \prime }\left(z\right)-\beta Y{}^{\prime \prime }\left(z\right),\\ F_{O}=-\nabla E_{O}. \end{array}$$

The partial differential equation of the number of iterations *c* is introduced to equation (6), and the following equation is obtained:

(8) $$\begin{array}{c} Y_{1}\left(a,b\right)=\alpha Y^{\prime \prime }\left(a,b\right)-\beta Y^{\prime \prime \prime \prime }\left(a,b\right)-\nabla E_{O}. \end{array}$$

When the position of the target contour is reached, the value of *Y*(*a*, *b*) is determined; then *Y*_1_(*a*, *b*)=0.

To solve the limited range of external force in the traditional deformable model algorithm, failures to converge to the concave edge, the image blur, and edge positioning accuracy decrease due to the increase of Gaussian standard deviation *α* and so forth; the GVF force field (equation (9)) is introduced, and the mapping range of the edge gradient is expanded with the aid of the vector diffusion equation (equation (10)) to improve the traditional variability model.

(9) $$\begin{array}{c} V\left(a,b\right)=\left[j\left(a,b\right),k\left(a,b\right)\right], \end{array}$$

(10) $$\begin{array}{c} \varepsilon =\iint \mu \left(j_{a}^{2}+j_{b}^{2}+k_{a}^{2}+k_{b}^{2}\right)+\left.|\nabla \int |{}^{2}|V-\nabla f|{}^{2}\right)da\;db, \end{array}$$

(11) $$\begin{array}{c} f=-E_{0}^{r},\;r\in \left\{1,2,3,4\right\}. \end{array}$$

From equation (10), the value of |∇∫| will affect the degree of *ε*; that is, when the value of *|*∇∫*|* is too small, the second-order partial derivative square term of the vector field determines the energy. When the value of *|*∇∫*|* is too large, the value of |*V* − ∇*f*|^2^ determines the magnitude of energy, and when *V*=∇*f*, the value of *ε* is the smallest. Since *V* in equation (9) includes both nonrotational and nondispersive components, the situation of nonconvergent concave edge contours caused by the opposite horizontal forces canceling each other out inside the concave edge is changed.

The following Euler equations are solved by variation:

(12) $$\begin{array}{c} \mu \nabla^{2}j-\left(j-f_{a}\right)\left(f_{a}^{2}+f_{b}^{2}\right)=0,\\ \mu \nabla^{2}k-\left(k-f_{a}\right)\left(f_{a}^{2}+f_{b}^{2}\right)=0. \end{array}$$

In the above equation, ∇^2^ represents the Laplace operator, and *j* and *k* are calculated by the discrete iterative value of the partial differential equation of the number of iterations. The differential equation is expressed as follows:

(13) $$\begin{array}{c} j\left(a,b,c\right)=\mu \nabla^{2}j\left(a,b,c\right)-\left[j\left(a,b,c\right)-f_{a}\left(a,b,c\right)\right]\left[f_{a}^{2}\left(a,b,c\right)+f_{b}^{2}\left(a,b,c\right)\right],\\ k\left(a,b,c\right)=\mu \nabla^{2}k\left(a,b,c\right)-\left[k\left(a,b,c\right)-f_{a}\left(a,b,c\right)\right]\left[f_{a}^{2}\left(a,b,c\right)+f_{b}^{2}\left(a,b,c\right)\right]. \end{array}$$

Through the above calculations, it is found that the GVF algorithm has poor convergence for the corners of the zero-order contour and the first-order contour, but it has a better convergence effect when processing concave contours with second-order continuity, based on which a dynamic vector field is constructed, and edge gradients are used in the edge area. The further algorithm improvement is made on the uniform area in the MRI image via the advantages of the GAF algorithm. The GAF algorithm equations before and after the improvement are expressed as follows:

(14) $$\begin{array}{c} Y_{1}\left(z,c\right)=\alpha Y^{\prime \prime }\left(z,c\right)-\beta Y^{\prime \prime \prime \prime }\left(z,c\right)+V, \end{array}$$

(15) $$\begin{array}{c} Y_{1}\left(z,c\right)=\alpha Y^{\prime \prime }\left(z,c\right)-\beta Y^{\prime \prime \prime \prime }\left(z,c\right)+P_{1}\left(\nabla f\right)V-P_{2}\left(\nabla f\right)\nabla f, \end{array}$$

(16) $$\begin{array}{c} P_{1}\left(\nabla f\right)=2-e^{|\nabla f|}, \end{array}$$

The introduction of *P*_1_(∇*f*) in equation (15) makes the GAF algorithm in the consistency area play a major role, and *P*_2_(∇*f*) is added to optimize the effect of *|*∇*f* in the edge area.

### 2.4. MRI Image Feature Acquisition and Vertebral Segmentation of Patients with Spinal Metastatic Tumors

**Figure 1:**
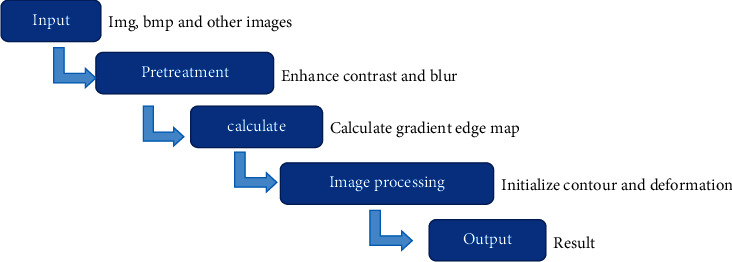
MRI image processing flowchart based on variable model algorithm.

The original MRI images of the experimental group were segmented according to the above-mentioned upgrade algorithm. The specific processing flowchart is shown in Figure 1.

To evaluate the vertebral segmentation effect of the improved variable model algorithm, the accuracy index (Dice coefficient (*D*)) was introduced, and the calculation is shown in equation (17). *D* expressed the proportion of the accurately segmented part in the segmentation result. The segmentation effect of five vertebrae in the MRI image of 50 patients in the experimental group was evaluated by calculating the pixel position when *D* was calculated, and the calculation results were averaged.

(17) $$\begin{array}{c} D=\frac{2|M\cap N|}{|M|+|N|}. \end{array}$$

### 2.5. Algorithm Evaluation Indexes

In this study, the recognition rate, sensitivity, and specificity indexes were used to evaluate the algorithm processing effect of the traditional variable model algorithm and the variable model improvement algorithm for MRI image segmentation of patients with spinal metastatic tumors. The equation for MRI image recognition rate is shown in equation (18), sensitivity in equation (19), and specificity in equation (20):

(18) $$\begin{array}{c} \text{recognition rate}=\frac{A+D}{A+B+C+D}, \end{array}$$

(19) $$\begin{array}{c} \text{sensitivity}=\frac{A}{A+D}\times 100\%, \end{array}$$

(20) $$\begin{array}{c} \text{specificity}=\frac{A}{A+C}\times 100\%, \end{array}$$

where *A* is the number of true positives, *B* is the number of false positives, *C* is the number of true negatives, and *D* is the number of false negatives.

### 2.6. Diagnosis Results of Primary Lesions in the Two Groups

The diagnosis results of the primary tumor of spinal metastasis in each patient in the experimental group and the control group were collected. These included but were not limited to lung cancer, breast cancer, prostate cancer, kidney cancer, liver cancer, cervical cancer, rectal cancer, colon cancer, bladder cancer, thyroid cancer, nasopharyngeal cancer, parotid ductal cancer, and ovarian cancer. The diagnosis results of the two groups were compared with the postoperative pathological diagnosis results. The accuracy of the MRI image analysis and diagnosis results of the two groups of patients was evaluated, and the diagnostic coincidence rate was calculated to measure the effect of improved variable model algorithm on MRI image diagnosis of patients with spinal metastatic tumors.

### 2.7. Statistical Methods

The test data processing was performed using SPSS 19.0. Mean ± standard deviation (*x* ± *s*) was how measurement data were expressed, and the comparison of the means between groups was performed by *t*-test. The count data was expressed by percentage (%), and the *χ*^2^ test was used. *P* < 0.05 indicated that the difference was statistically significant.

## 3. Results

### 3.1. MRI Image Vertebra Segmentation Effect Based on Improved Variable Model Algorithm

**Figure 2:**
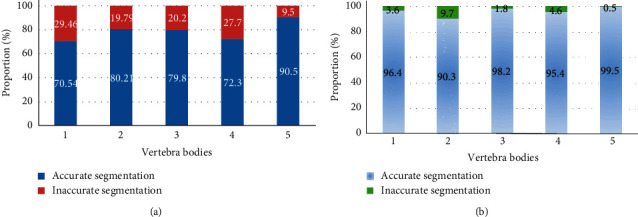
Comparison of *D* values of the two algorithms. (a) The traditional variable model algorithm; (b) the improved variable model algorithm.

It was found that the values of *P*_1_(∇*f*) and *P*_2_(∇*f*) had a great influence on the effect of MRI image processing when the active contour of the MRI image was processed. It was also found that the improved variable model algorithm had the best segmentation effect on the spine when *P*_1_(∇*f*)=0.8 and *P*_2_(∇*f*)=0.5 in the processing of MRI images for spinal metastatic tumors. In addition, the segmentation effect was the best when *μ*=0.06 in equation (10). The segmentation of the five vertebrae in the MRI images of the experimenter before and after the improvement of the variable model algorithm was summarized and counted, and the segmentation effect of the vertebrae in the MRI images was evaluated according to the average *D* value of the segmentation of the vertebrae in the MRI images, and the comparison of the average *D* values of the segmentation of the five vertebrae in the MRI images of the specific two algorithms was shown in Figure 2. As can be concluded from the figure, the average *D* values of the conventional variable model algorithm for segmentation of the five vertebrae in the MRI images of the experimenter were 71%, 80%, 80%, 71%|, and 91%, respectively, while the average *D* values of the variable model improvement algorithm for segmentation of the five vertebrae in the MRI images of the experimenter were 96%, 90%, 98%, 95%, and 100%, respectively, and the results showed that the variable model improvement algorithm was significantly better than the traditional algorithm in segmenting vertebrae in MRI images of patients in the experimental group, and the difference was statistically significant (*P* < 0.05).

### 3.2. Comparison of MRI Image Processing Effect between Traditional Variable Algorithm and Improved Variable Model Algorithm

**Figure 3:**
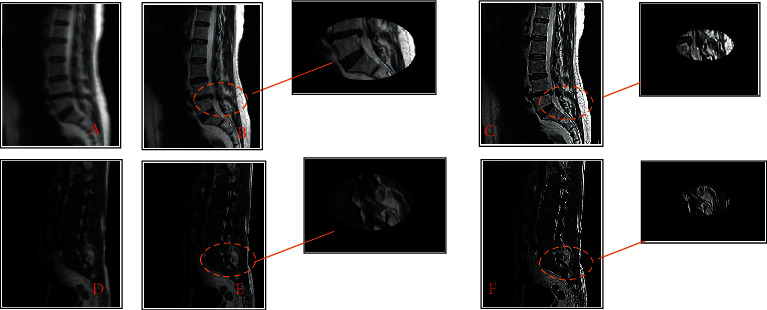
Comparison of MRI images of patients with spinal metastatic tumors processed by different algorithms.

Figure 3 shows the MRI images of two patients with spinal metastatic tumors. Figures 3(a)–3(c) show MRI images of a 56-year-old female patient. Figures 3(d)–3(f) show MRI images of a 49-year-old male patient. Figures 3(a) and 3(d) show conventional MRI images, while Figures 3(b) and 3(e) show MRI images processed by the traditional variable model algorithm. Figures 3(c) and 3(f) show MRI images processed by the improved variable model algorithm. The clarity of MRI was improved after repeated iterative segmentation of MRI images by variable model algorithm, and the recognition speed of the patient's lesions was greatly improved. It realized rapid and accurate positioning, which can diagnose spinal metastatic tumors faster and more accurately.

MRI images showed that patients with spinal metastatic tumors had infiltrated and destroyed bone marrow tissue of the vertebral body due to metastatic tumors. The replacement of normal bone marrow cells by tumor tissue in patients with spinal metastatic tumors caused prolonged T1 and T2 relaxation times. According to the degree of tumor erosion and destruction, T1WI showed limited or diffuse low signal, which was prominent in the background of normal bone marrow signal. Metastases in T2WI were often fused with the surrounding normal adipose bone marrow, making it difficult to distinguish.

### 3.3. Comparison of MRI Image Evaluation Indexes between Traditional Variable Model Algorithm and Improved Variable Model Algorithm

**Figure 4:**
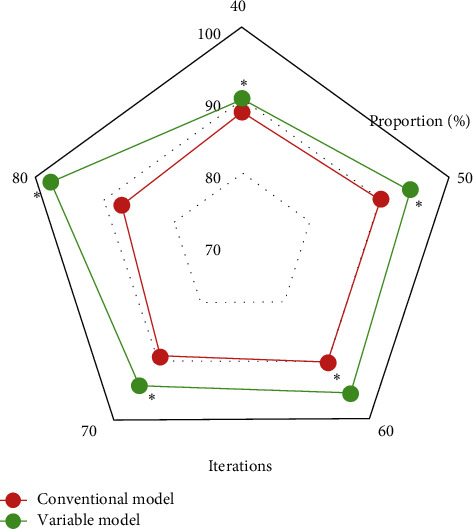
Comparison of MRI image recognition rate of the two algorithms.

**Figure 5:**
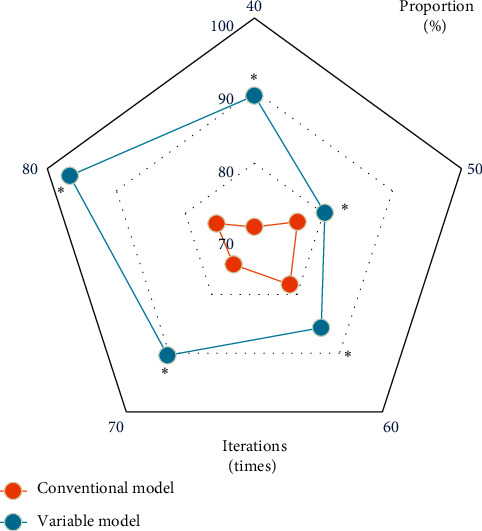
Comparison of MRI image sensitivity of the two algorithms.

**Figure 6:**
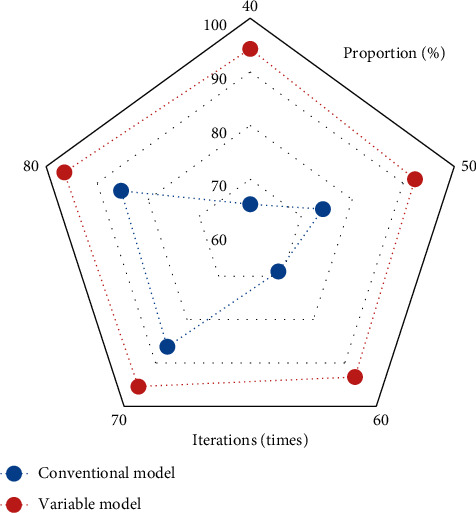
Comparison of MRI image specificity of the two algorithms.

Figures 4–6 represent the comparison of recognition rate, sensitivity, and specificity indexes of the traditional variable model algorithm and the variable model improvement algorithm. As can be concluded from the figures, the recognition rates of MRI image segmentation of the traditional variable model algorithm at 40, 50, 60, 70, and 80 iterations were 88.31%, 90.24%, 90.31%, 89.32%, and 87.54%, respectively, and the sensitivity of MRI image segmentation was 71.23%, 76.25%, 78.31%, 74.88%, and 75.48%, and MRI image segmentation specificity was 65.32%, 74.25%, 68.89%, 86.27%, and 85.31%, respectively; meanwhile the MRI image segmentation recognition rates of the variable model improvement algorithm at 40, 50, 60, 70, and 80 iterations were 90.23%, 94.51%, 95.6%, 94.23%, and 97.89%, the image segmentation sensitivity was 89.32%, 80.21%, 85.65%, 90.37%, and 96.75%, and the MRI image segmentation specificity was 94.3%, 92.3%, 93.21%, 95.43%, and 96.45%, respectively. It can be concluded that the variable model improvement algorithm can improve the recognition rate, sensitivity, and specificity of MRI images of spinal metastatic tumors compared with the traditional variable model algorithm, and the comparison of the three indexes showed significant differences with statistical significance (*P* < 0.05). Therefore, the variable model improvement algorithm after the introduction of GVF force field can significantly improve the recognition rate, sensitivity, and specificity of lesion features in MRI images of spinal metastatic tumors, which is more beneficial to the clinical diagnosis of this disease.

### 3.4. Comparison of Diagnostic Coincidence Rate between Improved Variable Model Algorithm and Traditional Manual MRI Image Analysis

**Figure 7:**
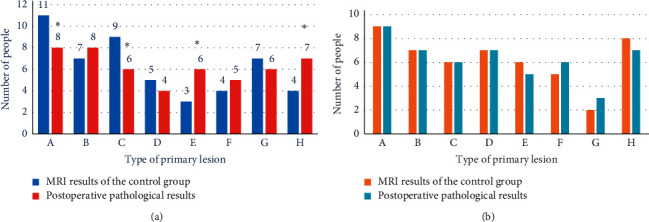
Comparison of primary lesions between the two groups of patients. (a) The comparison between the diagnosis results of control group and the postoperative pathological diagnosis results. (b) The comparison between the diagnosis results of experimental group and the postoperative pathological diagnosis results. A, B, C, D, E, F, G, and H represent lung cancer, breast cancer, prostate cancer, cervical cancer, rectal cancer, colon cancer, kidney cancer, and hepatocellular carcinoma, respectively. ^*∗*^ indicates a statistically significant difference with postoperative pathological findings (*P* < 0.05).

**Figure 8:**
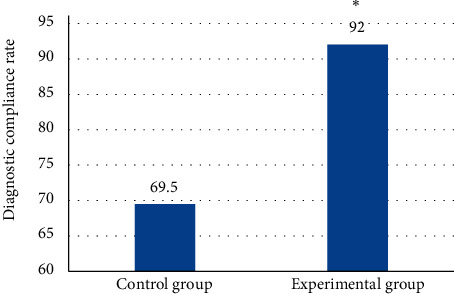
Comparison of the diagnosis coincidence rate between the two groups of patients. ^*∗*^indicates a statistically significant difference with postoperative pathological findings (*P* < 0.05).

The diagnosis results of spinal metastatic tumors on MRI images of the two groups of patients were summarized. The diagnosis of primary lesions of spinal metastatic tumors and the postoperative pathological diagnosis of the two groups of patients were made into a comparison chart (Figure 7). As shown in Figure 7, there are some differences between the diagnostic results of conventional manual MRI image analysis methods for lung cancer, breast cancer, prostate cancer, cervical cancer, rectal cancer, colon cancer, kidney cancer, and hepatocellular carcinoma in the control group and the postoperative pathological diagnoses, among which the diagnostic results of conventional manual MRI image analysis for metastatic tumors of the spine with primary lesions belong to lung cancer, prostate cancer, rectal cancer, and hepatocellular carcinoma. There was a significant difference (*P* < 0.05). The results in Figure 8 show that the diagnostic compliance rate of conventional manual MRI image analysis was 69.5%, and the diagnostic compliance rate of MRI images processed based on the variable model algorithm was 92%. Compared with the traditional manual MRI image diagnosis, the MRI image diagnosis based on the variable model algorithm was more accurate for the determination of the primary lesion location, and the diagnostic compliance rate was significantly improved. There was a significant difference (*P* < 0.05).

## 4. Discussion

The spine is the central axis of the human body and is a common site for bone tumors. There are many types of tumors, which will seriously affect the quality of life of patients. It can cause spinal pain, radiating pain, weakness of the limbs, and even paraplegia in severe cases [15]. Due to the complex anatomical structure of the spine and obvious overlap, there will be missed and misdiagnosed clinical imaging diagnosis. MRI is one of the effective diagnostic methods for spinal metastatic tumors. Its soft tissue resolution and sensitivity to intramedullary lesions are significantly higher than those of plain radiographs and CT. Moreover, it can detect abnormal signals in the early stage of vertebral disease, showing significant advantages in the diagnosis of paravertebral soft tissue masses, intraspinal canal invasion, and spine skipping lesions. Coupled with its own nonradiation, it is especially suitable for imaging the spine [16]. However, the quality of MRI images is affected by the image's signal-to-noise ratio, image contrast, artifacts, image spatial resolution, and inspection conditions, which in turn affects the effectiveness of disease diagnosis [17]. Therefore, improving the quality of MRI images is a difficult problem in current research.

In recent years, to improve the image quality and the recognition rate of characteristic lesions in MRI images, improved variable model algorithms were introduced in a number of studies to realize effective segmentation of MRI images [18]. The algorithm reduces human-computer interaction by presetting the initial position of the model and introduces an energy function to improve the segmentation effect of the segmentation algorithm. The interactive and variable segmentation of MRI images is completed, combined with the typical characteristics of the disease and the problems in conventional image segmentation [19]. At the same time, the use of mathematical algorithm models to analyze MRI images to assist physicians in diagnosing the spread of metastatic lesions in the spine is a hot topic of research. In this research, an MRI image analysis model for spinal metastatic lesions is designed based on the variable model algorithm and applied it to 50 cases of spinal metastatic lesions in the experimental group. The results showed that MRI images processed by the variable model algorithm were more effective than traditional variable model images in the diagnosis of spinal metastatic lesions. The mean *D* value of vertebral segmentation of spinal metastases was significantly higher with statistically significant differences (*P* < 0.05) compared with the traditional variable model algorithm, and the recognition rate, sensitivity, and specificity indexes of MRI images were significantly better than those of the traditional variable model algorithm. In the comparison of the MRI images, it was found that the variable model algorithm was more accurate in identifying the focal points of patients with metastatic tumors in the spine, and the accuracy of the MRI images based on the variable model algorithm was significantly improved when compared with the traditional MRI images. The experimental results were in agreement with those of Namías et al. [20]. In short, MRI image analysis based on variable model algorithm was of great adoption potential in the clinical diagnosis of spinal metastatic tumors. This research is the clinical adoption and promotion of improved variable model algorithm in MRI image segmentation and disease diagnosis.

## 5. Conclusion

In this work, an improved variable model algorithm for spinal metastatic tumors was designed based on the traditional variable model algorithm and was applied to the MRI image analysis of 50 patients in the experimental group. It was found that the improved variable model algorithm can significantly improve the diagnostic coincidence rate of MRI images compared with the control group, and the identification of lesion sites was more accurate. However, the selection of patient samples in this study is small and the source is single, which makes this work does not complete enough data collection on the primary site of spinal metastatic tumors. Therefore, it fails to discuss the different manifestations of spinal metastatic tumors of different types of primary sites in detail, and it is impossible to verify the influence of these characteristics on the accuracy of prediction. In the future, we will consider increasing the sample size of patients with spinal metastatic tumors and further adopt a multicenter collaborative analysis method for research. ln conclusion, the results provide a good theoretical basis for the adoption of variable model algorithm in MRI imaging diagnosis of clinical spinal metastatic tumors.

## Data Availability

The data used to support the findings of this study are available from the corresponding author upon request.
